# Research Progress in the Preparation of Lactide

**DOI:** 10.3390/polym18121484

**Published:** 2026-06-12

**Authors:** Meiqi Tian, Yingjian Zhou, Junhao Wang, Ziqi Cai, Zhipeng Li, Zhengming Gao

**Affiliations:** 1 Department of Chemical Engineering, Beijing University of Chemical Technology, Beijing 100029, China; mqtian2000@163.com (M.T.); caiziqi@mail.buct.edu.cn (Z.C.); gaozm@buct.edu.cn (Z.G.); 2 China Kunlun Contracting & Engineering Corporation, Beijing 100037, China; zhouyingjian@cnpc.com.cn

**Keywords:** lactide synthesis, polylactic acid, catalytic systems, one-step process, two-step process, process intensification

## Abstract

Driven by the growing demand for sustainable polymers, polylactic acid (PLA) has attracted increasing attention due to its renewable origin and biodegradability. Lactide, the key cyclic monomer for PLA production via ring-opening polymerization (ROP), plays a decisive role in determining the molecular weight, stereoregularity, and final performance of PLA materials. However, current lactide synthesis processes still face significant challenges, including competing side reactions under high-temperature and high-vacuum conditions, difficulties in controlling stereochemical purity, and relatively high energy consumption. In this review, recent advances in lactide synthesis are systematically analyzed by examining the two principal industrial routes: the one-step process based on the direct dehydration–cyclization of lactic acid (LA), and the two-step process involving prepolymerization of LA followed by depolymerization/cyclization of oligomeric intermediates. The reaction mechanisms, key intermediates, and major side reactions—including racemization, transesterification, and deep polycondensation—are discussed, together with the regulatory roles of catalytic systems and reaction–separation coupling strategies. Comparative analysis reveals that the one-step route offers advantages in process integration and potential energy efficiency, whereas the two-step route provides superior control over stereochemical purity and process stability. Future research directions focusing on green catalysts, process intensification, and sustainable lactide production are also highlighted.

## 1. Introduction

With the rapid growth of the global population, the large-scale production, use, and disposal of polymeric materials derived from fossil resources such as petroleum and coal have imposed increasing ecological pressure, thereby raising widespread awareness of the urgency of environmental protection [1,2]. In this context, the development of polymeric materials derived from renewable resources and featuring inherent biodegradability has become a central focus in materials science and environmental engineering [3]. Among the various candidates, polylactic acid (PLA) is widely regarded as one of the most promising biobased polymers due to its abundant renewable feedstocks, well-established green synthesis routes, favorable mechanical properties, and its ability to degrade under industrial or natural conditions [4,5]. The rapid expansion of PLA applications in packaging, biomedical devices, fibers, and additive manufacturing further underscores its potential to reduce reliance on petrochemical resources and mitigate environmental burdens [6,7,8,9].

Despite these advantages, the performance and application scope of PLA remain highly dependent on its upstream synthesis pathways and catalytic systems [10]. PLA can be produced either by the direct polycondensation of lactic acid (LA) or via ring-opening polymerization (ROP) using lactide as an intermediate. Among these routes, the stereochemical purity of lactide and the control of side reactions during polymerization are widely recognized as key determinants of the molecular weight, stereoregularity, and final properties of PLA [11,12,13]. From an industrial perspective, limitations associated with direct polycondensation, such as unfavorable equilibrium constants, restricted dehydration efficiency, and difficulties in achieving high molecular weights, have led to the establishment of the lactide-mediated ROP route as the dominant technological framework for large-scale PLA production, which has now been successfully industrialized worldwide [14]. Accordingly, a deeper understanding of the synthesis of PLA from monomer production to polymerization, together with the development of more controllable, robust, and application-oriented manufacturing processes, is of critical importance for improving material performance and expanding the practical utility of PLA.

**Figure 1:**
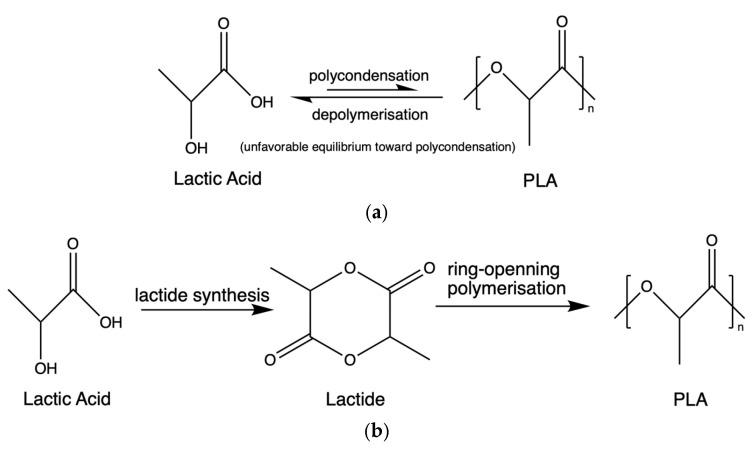
Synthetic pathways for the preparation of PLA: (**a**) direct polycondensation of LA; (**b**) lactide-mediated ROP.

The polymerization of LA generally follows two major pathways, as illustrated in Figure 1: direct polycondensation and the lactide-mediated ROP route [15]. In the direct polycondensation approach, LA is polymerized through step-growth condensation; however, this process is intrinsically limited by an unfavorable equilibrium constant, inefficient water removal, and the formation of secondary products such as lactide, cyclic oligomers, and other low-molecular-weight species. As a result, the molecular weight of the resulting PLA is typically low (Mn ≈ 10^3^ g/mol), rendering this route unsuitable for high-performance applications [16].

Consequently, industrial PLA production predominantly relies on the lactide-ROP route, in which LA is first polycondensed into low-molecular-weight oligomers, subsequently depolymerized to lactide, and finally converted into high-molecular-weight PLA via ROP [17]. Within this technological framework, lactide serves as the critical intermediate linking LA to PLA, and its molecular structure and purity directly determine the attainable molecular weight, stereoregularity, and ultimate material performance of the polymer [18,19,20]. Notably, even when lactide purity is well controlled, the reaction temperature and polymerization medium employed during ROP can still significantly influence stereochemical retention and the occurrence of side reactions, thereby imposing stringent requirements on the precise control of PLA microstructure [21]. Industrial experience further demonstrates that only lactide monomers with high chemical purity and well-defined optical composition enable the robust production of PLA with molecular weights exceeding 100 kg/mol and tunable properties via ROP, a conclusion that has been validated across multiple industrial-scale continuous polymerization processes [22].

**Figure 2:**
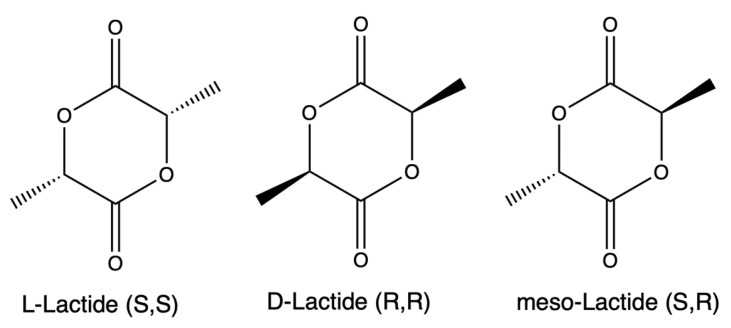
Three stereochemical isomers of lactide.

Lactide (3,6-dimethyl-1,4-dioxane-2,5-dione) is the dehydrated cyclic dimer of LA. Owing to the presence of two enantiomeric forms of LA, lactide correspondingly exists in three stereochemical isomers: L-lactide (S,S), D-lactide (R,R), and meso-lactide (S,R), as illustrated in Figure 2. In addition, equimolar mixtures of D- and L-lactide form racemic lactide (rac-lactide or D,L-lactide), further expanding the stereochemical diversity of lactide monomers [23,24]. Among these stereoisomers, L-lactide is the most readily available and widely used due to its derivation from naturally abundant L-LA [15].

Advanced characterization techniques, particularly nuclear magnetic resonance spectroscopy, have demonstrated that these stereoisomeric lactides can be distinctly incorporated during ROP, with their stereochemical sequences being directly reflected in the chain microstructure of the produced PLA [25]. Subsequent studies have established that variations in lactide stereochemical composition exert a pronounced influence on the stereoregularity and crystallization behavior of PLA chains, ultimately leading to significant differences in thermal properties such as the glass transition temperature and melting behavior [26]. These effects are closely associated with the retention and transfer of stereochemical information during the ROP process and have been systematically elucidated across a range of controlled polymerization systems [27].

Despite the long-standing use of lactide in both industrial production and laboratory research, its synthesis remains subject to significant technical limitations. In industrial practice, lactide production typically involves multiple sequential operations, including prepolymerization, continuous depolymerization, and distillation-based purification. These steps are highly sensitive to temperature control, catalyst selection, residence time, and trace water content. Such process complexity not only results in high energy consumption and stringent equipment requirements, but also makes it difficult to maintain stable product yield and optical purity during long-term operation [28,29]. Recent studies have further revealed that the individual reaction steps involved in lactide synthesis are strongly coupled, leading to an exceptionally narrow operational window. Minor fluctuations in process conditions can readily amplify side reactions or disrupt stereochemical control, thereby substantially increasing the difficulty of process scale-up and long-term continuous operation [30]. The associated high production cost has consequently emerged as a critical bottleneck, limiting further cost reduction and the large-scale deployment of PLA production [31].

From an academic perspective, although the decisive role of lactide structure in determining PLA properties has been well established, a systematic understanding of its formation pathways, the origins of side reactions, and the mechanisms governing stereochemical selectivity remains incomplete [15,32]. Available studies indicate that both the ring structure and stereochemical composition of lactide are highly sensitive to reaction conditions during its synthesis and subsequent handling. Such variability not only deteriorates product purity but also significantly complicates the precise control of the overall reaction process [33]. With the increasing demand for greener, more selective, and sustainable polymer production, the development of environmentally benign catalytic systems and controllable reaction pathways has therefore emerged as a central research direction in the field of lactide synthesis [34,35].

Taken together, the central challenge in contemporary lactide synthesis does not lie in whether conversion can be achieved, but rather in how high yield, optical purity, and long-term operational stability can be simultaneously maintained under conditions characterized by high temperatures, competing reaction pathways, and complex mass-transfer limitations. While such trade-offs can often be mitigated at the laboratory scale through fine-tuned control of reaction parameters, they are substantially amplified during industrial continuous scale-up, where minor fluctuations readily translate into pronounced losses in selectivity and stability. As a result, this intrinsic contradiction has emerged as a critical bottleneck hindering further cost reduction and the advancement of lactide-based processes toward high-end and large-scale applications.

Driven by the growing demand for PLA and the broader strategic shift toward sustainable materials, extensive research efforts have been devoted to elucidating the reaction mechanisms, developing advanced catalytic systems, suppressing side reactions, and enabling continuous processes for lactide production. At the level of lactide synthesis itself, distinct from the downstream PLA polymerization pathways, the production technologies can generally be divided into two representative strategies according to whether a preformed oligomeric intermediate is involved [15,32].

The first is the direct one-step dehydration–cyclization route, in which lactide is produced directly from LA through dehydration and cyclization without going through a separate low-molecular-weight oligomer/prepolymer stage. The second is the two-step prepolymerization-depolymerization route, where LA is first converted into prepolymers with defined molecular weight and stereochemical characteristics, followed by controlled depolymerization to lactide.

In this review, these two representative strategies are taken as the central framework to systematically examine their underlying reaction mechanisms, technological evolution, inherent bottlenecks, and future optimization directions. By integrating insights from catalysis, reaction engineering, and process intensification, this work aims to provide a comprehensive and coherent understanding of efficient, green, and controllable lactide synthesis.

## 2. One-Step Synthesis of Lactide

### 2.1. Process Principle

The one-step synthesis of lactide refers to a reaction route in which LA is used as the feedstock and directly converted into lactide through dehydration and cyclization in the presence of a suitable catalyst. This process emphasizes the transformation of LA units into the six-membered cyclic lactide structure without involving an independent prepolymer preparation stage [36].

**Figure 3:**
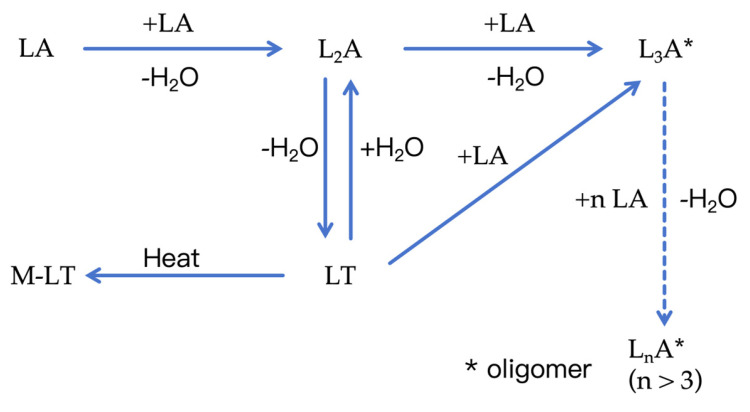
Representative reaction pathways and competing side reactions for lactide formation from LA via a dimeric intermediate. Redrawn based on Ref. [37].

From the perspective of the reaction pathway, LA molecules are first adsorbed and activated on the catalyst surface or within the pore environment, followed by dehydration between two LA units to form the LA dimeric intermediate L_2_A, which can further undergo cyclization to generate lactide. In this context, L_2_A is more appropriately understood as a reactive intermediate involved in the direct cyclization process. After its formation, L_2_A may further undergo dehydration–cyclization to produce lactide, rather than serving as a preformed and accumulated low-molecular-weight prepolymer. Studies on continuous gas-phase one-step processes, liquid-phase zeolite-catalyzed systems, and theoretical calculations based on gas-phase models all support the direct dehydration–cyclization pathway of LA → L_2_A → lactide [37,38,39]. However, under practical reaction conditions, L_2_A may either undergo further dehydration–cyclization to form lactide or continue to condense with LA to generate higher oligomers. In addition, the generated lactide may undergo side reactions such as hydrolysis or thermally induced isomerization. The representative pathways are illustrated in Figure 3 [37].

From the perspective of reaction control, the dehydration condensation of LA and the cyclization of L_2_A in the one-step process are first driven by thermodynamic dehydration effects; that is, timely removal of water favors the shift in the condensation/cyclization equilibrium toward lactide formation. However, the final selectivity toward lactide is not determined solely by thermodynamic equilibrium, but is mainly governed by kinetic competition among different reaction pathways. Specifically, L_2_A may either cyclize to form lactide or continue to undergo oligomerization, while the generated lactide may further experience hydrolysis or isomerization. Therefore, the key to the one-step process lies in regulating catalyst structure, water removal, and product desorption so that the rates of L_2_A cyclization and timely lactide removal prevail over those of side reactions such as oligomerization, hydrolysis, and isomerization [37,38,40].

### 2.2. Process Characteristics

The process characteristics of one-step lactide synthesis are mainly reflected in its direct process flow and the tight coupling of reaction, dehydration, and product separation. Since the direct dehydration–cyclization of LA is highly sensitive to water content and product stability, process optimization should focus not only on improving LA conversion, but also on simultaneously enhancing water removal, suppressing lactide hydrolysis, and reducing the formation of oligomeric by-products [40,41].

In liquid-phase one-step systems, water removal is a key factor affecting reaction progression and product quality. Liu et al. reported that, in an H-Beta/toluene system, toluene can remove water through azeotropic distillation, condensation, and phase separation; however, residual water in refluxing toluene may still induce lactide hydrolysis. By enhancing toluene-water phase separation with CaCl_2_, the water content in the organic phase can be further reduced, thereby improving lactide yield [40]. Similarly, in the direct synthesis of lactide from LA over zeolite Beta, Leung et al. obtained high-purity lactide through successive treatments including Dean–Stark dehydration, molecular-sieve drying of refluxing toluene, and dry recrystallization, further demonstrating the importance of deep dehydration and subsequent purification for product quality in one-step processes [41]. In addition, the study by Ma et al. on F-modified Beta zeolites showed that increasing pore hydrophobicity helps reduce water adsorption near acidic sites, thereby promoting local water removal and suppressing lactide hydrolysis. This indicates that liquid-phase one-step synthesis is also influenced by the water microenvironment within catalyst pores [42].

However, water does not merely act as a reactant in lactide hydrolysis, and its role may also be system-dependent. Theoretical calculations on Sn-Beta zeolites have shown that the participation of one water molecule in the L_2_A cyclization process can lower the reaction energy barrier, suggesting that water may participate in proton transfer and promote cyclization within specific catalytic microenvironments. Nevertheless, excessive water still promotes hydrolysis and oligomeric side reactions [43].

Gas-phase one-step synthesis is typically carried out continuously in a fixed-bed reactor. After being preheated and vaporized, the aqueous LA feed enters a bed packed with a solid catalyst, where dehydration–cyclization proceeds under gas-phase conditions, followed by cooling or condensation to collect crude lactide. The study by Park and Chang showed that the preheating temperature, reaction temperature, carrier gas flow rate, and space velocity all affect LA conversion, lactide selectivity, and oligomer formation. The introduction of a glass-bead buffering layer can improve gas distribution and reduce oligomer deposition [35]. The SnO_2_-SiO_2_ gas-phase system reported by Upare et al. also demonstrated that shortening the high-temperature residence time and promoting timely product desorption are beneficial for improving lactide selectivity and reducing the risk of its further conversion into oligomers [37].

From the perspectives of techno-economic and life-cycle analyses, the one-step gas-phase process shows potential for reducing process complexity, lowering separation burdens, and improving sustainability. Heo et al. established a conceptual process flowsheet based on the one-step gas-phase route and compared its conversion cost and global warming potential. Their results indicated that this route exhibits certain advantages in both economic and environmental performance. However, its overall performance is highly dependent on the operating conditions of the reaction section, particularly because reaction temperature and crude lactide purity can significantly influence the downstream purification load [44].

In terms of the relative contributions of competing pathways under different operating conditions, in liquid-phase one-step systems, when the water content is relatively high or dehydration of the refluxing organic phase is insufficient, lactide hydrolysis and the continued condensation of LA/oligomers are more likely to become the dominant side-reaction pathways, resulting in decreased selectivity toward the target cyclization reaction. By contrast, when the local water concentration is reduced through azeotropic dehydration, phase-separation-assisted drying, or regulation of hydrophobic pore environments, the contribution of further dehydration–cyclization of L_2_A to lactide is correspondingly enhanced [40,41,42]. For gas-phase one-step systems, elevated temperature is beneficial for LA vaporization and dehydration–cyclization. However, if the residence time is excessively prolonged or product desorption is not sufficiently rapid, the generated lactide may further undergo side reactions and be converted into oligomeric species. Conversely, under conditions of short residence time, appropriate space velocity, and rapid condensation, cyclization and product removal become the dominant processes, thereby favoring improved lactide selectivity [35,37,44]. Therefore, although dehydration and water removal provide an important thermodynamic driving force for lactide formation, the product distribution in the one-step process is mainly governed by kinetic competition among L_2_A cyclization, oligomerization, lactide hydrolysis, and thermally induced isomerization.

**Figure 4:**
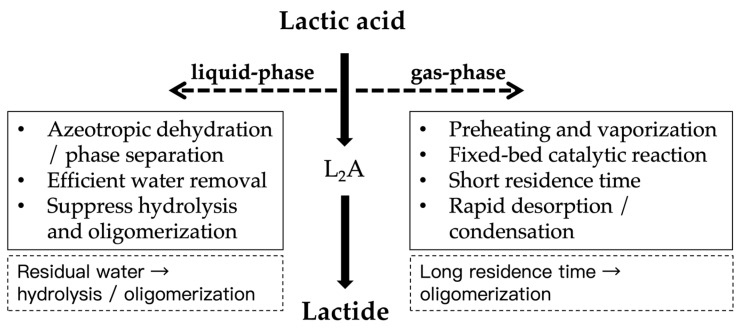
Schematic illustration of the process characteristics of one-step lactide synthesis.

Figure 4 summarizes the major pathways involved in one-step lactide synthesis, including both liquid-phase and gas-phase one-step routes, while also illustrating their corresponding competing pathways.

Overall, the advantages of the one-step process arise from the synergistic integration of LA conversion, dehydration, product removal, and subsequent purification. For liquid-phase systems, the key lies in enhancing water removal and suppressing lactide hydrolysis, whereas for gas-phase systems, the critical issue is the coordinated matching among LA vaporization, heat and mass transfer within the catalyst bed, residence time, and rapid product condensation. Therefore, although the one-step process shows potential for process simplification and greener production, its stable operation still relies on the integrated optimization of reaction conditions, water management, and separation processes [40,41,42,44].

### 2.3. Typical Catalyst Systems in One-Step Lactide Synthesis

Based on catalyst structure and the characteristics of active centers, the catalyst systems used for one-step lactide synthesis can be broadly classified into three categories: zeolitic solid acid catalysts, Lewis acid heterogeneous catalysts, and organic-framework-based confinement cooperative catalysts. Although all three types of systems aim to promote the direct dehydration–cyclization of LA, their catalytic functions emphasize different aspects of the reaction process.

#### 2.3.1. Zeolitic Solid Acid Catalysts

Zeolitic solid acids represent one of the most typical catalyst systems for one-step lactide synthesis, with representative examples including H-Beta, ZSM-5, and their modified zeolitic derivatives. The function of these catalysts is not limited to the provision of acidic sites. More importantly, through the synergistic interaction between acid centers and regular pore structures, they promote the dehydration condensation of LA units and regulate the cyclization of the LA dimeric intermediates toward six-membered cyclic lactide. Liu et al. achieved the direct conversion of LA to lactide using H-Beta zeolite as the catalyst in a toluene system, indicating that Beta-type zeolites can provide both acidic sites and a pore-confined environment for one-step reactions [40].

On this basis, the optimization of zeolitic catalysts has gradually expanded from conventional acidity regulation to the design of pore architecture, diffusion pathways, and local reaction environments. Ma et al. regulated the pore hydrophobicity of Beta zeolites through F modification, thereby improving the selectivity toward lactide formation while maintaining the acidic function of the catalyst [42]. Using ZSM-5 nanosheets as an example, Zhang et al. demonstrated that reducing the b-axis thickness can enhance the accessibility of Brønsted acid sites and improve the mass-transfer efficiency of reactants and products within the pores, thereby promoting the cyclization conversion of L_2_A to lactide [38]. These studies indicate that the performance of zeolitic solid acid catalysts depends on the coordinated matching among acidic sites, pore size, diffusion pathways, and local reaction environments, rather than on acid strength or acid amount alone.

#### 2.3.2. Lewis Acid Heterogeneous Catalysts

Lewis acid heterogeneous catalysts represent another important class of catalyst systems in one-step lactide synthesis, with representative examples including metal heteroatom-containing Sn-Beta zeolites and SnO_2_-SiO_2_ nanocomposite catalysts. Compared with zeolitic solid acid systems dominated by Brønsted acid sites and pore confinement, this type of catalyst places greater emphasis on the adsorption and activation of carbonyl groups in LA or LA dimeric intermediates by metal Lewis acid centers.

Although Sn-Beta possesses a Beta zeolite framework, its catalytic function mainly originates from the Lewis acid sites formed by framework Sn species. Therefore, it can be regarded as a metal-heteroatom zeolite Lewis acid catalyst. Xu et al. demonstrated that open Sn sites in Sn-Beta, namely HO-Sn-(OSi)_3_ and adjacent Si-OH groups, are crucial for enhancing catalytic activity. After these open sites were shielded by Na^+^ exchange, the reaction rate decreased markedly. DFT calculations further showed that the interaction between the substrate and the open Sn sites was weakened, accompanied by an increase in the reaction energy barrier [43].

SnO_2_-SiO_2_ is a representative metal oxide catalytic system for gas-phase one-step synthesis. The SnO_2_-SiO_2_ catalyst reported by Upare et al. enabled the direct conversion of LA to lactide under continuous atmospheric-pressure conditions and exhibited high yield, optical selectivity, and stability. In this system, SnO_2_ shows relatively strong adsorption toward LA but weak adsorption toward lactide, which is favorable for LA activation and timely product desorption, thereby reducing the risk of further oligomerization or degradation [37].

#### 2.3.3. Organic-Framework-Based Confinement Cooperative Catalysts

In addition to inorganic zeolites and metal oxides, organic framework materials such as COFs provide new perspectives for the design of one-step lactide synthesis catalysts. These materials feature tunable pore structures and designable functional groups, enabling the direct cyclization of LA to be cooperatively regulated through confinement effects and multifunctional active sites.

The OH-COOH-COF reported by Zhao et al. can catalyze the direct conversion of L- LA to L-lactide under relatively mild conditions. The ordered pores of this catalyst contain functional sites such as carboxyl and ketone groups, which promote the oriented dimerization and cyclization of LA molecules through hydrogen-bonding interactions and pore confinement. Comparative experiments showed that neither functional groups alone nor the framework structure alone were sufficient to achieve high catalytic performance. Instead, the appropriate pore size, ordered framework structure, and synergistic interaction among multifunctional sites jointly determine its selectivity and ability to suppress oligomerization [45].

The reported performance of these representative catalysts in terms of lactide yield, selectivity, optical purity, and reaction conditions is summarized in Table 1.

Overall, zeolitic solid acid catalysts emphasize the matching between acidic sites and the pore environment, Lewis acid heterogeneous catalysts focus on the balance between Lewis acid activation and product desorption, whereas organic-framework-based catalysts highlight the designability of pore structures and functional groups. Collectively, these systems indicate that catalyst design for one-step lactide synthesis is evolving from single acidity regulation toward the synergistic optimization of acidity, pore architecture, water microenvironment, and product desorption behavior.

## 3. Two-Step Synthesis of Lactide

### 3.1. Process Principle

The two-step process remains the dominant industrial route for lactide production. Its fundamental principle lies in decoupling LA polycondensation from depolymerization–cyclization by implementing these steps in a sequential manner. Through spatial and temporal separation, each reaction stage can be conducted under its respective optimal conditions, thereby enabling precise control over reaction pathways and stereochemical behavior [15,32,46].

In this process, LA is first subjected to continuous dehydration and polycondensation under moderate temperatures to form oligomeric PLA. The primary objective of this stage is to construct a linear oligomer system with well-defined chain ends, controlled molecular architecture, and extremely low residual water content. The average chain length of the oligomers, their terminal chemical environment, and the remaining water content directly govern the kinetics of the subsequent depolymerization step, the preferred cyclization pathway, and the preservation of stereochemical integrity in the lactide product [46,47].

**Figure 5:**
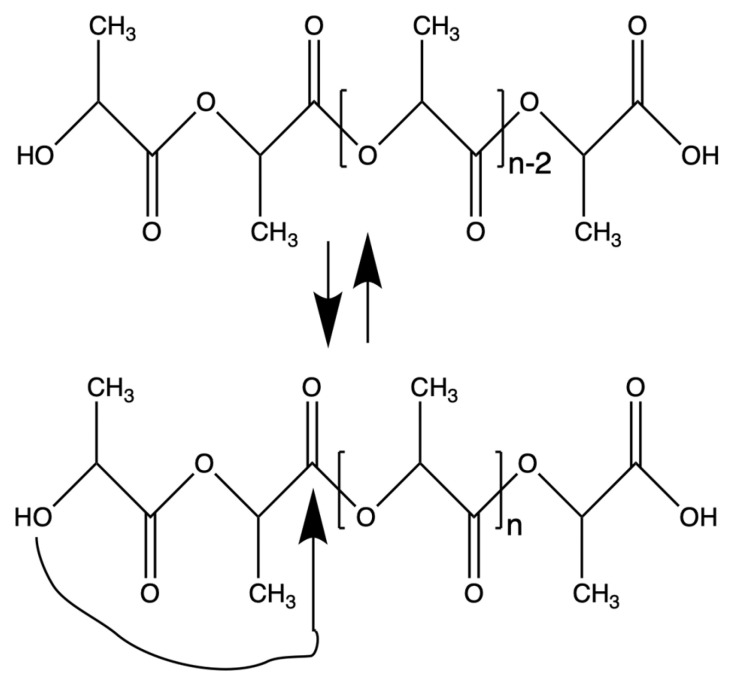
Schematic illustration of lactide ring formation. Redrawn based on Ref. [48].

Subsequently, the obtained oligomeric PLA undergoes intramolecular back-biting and lactonization reactions at a higher temperature and under high vacuum conditions. That is, the hydroxyl group at the chain end conducts a nucleophilic attack on the adjacent ester bond, forming a six-membered ring structure of lactide, as shown in Figure 5. The formed lactide is rapidly removed from the reaction zone through synchronous distillation [44,48,49]. This stage essentially constitutes a highly coupled reaction–separation process involving thermal depolymerization and cyclization, in which the reaction rate, equilibrium shift, and extent of side reactions are jointly influenced by temperature, vacuum level, evaporation efficiency, and trace amounts of water present in the system [50,51].

From a thermodynamic perspective, LA dehydration polycondensation is a reversible step-growth condensation reaction that is significantly affected by water content. Harshe et al. estimated the equilibrium constant of LA polycondensation in a closed system to be approximately 10 and defined “complete water removal” as the ideal irreversible polycondensation limit. This indicates that ideal dehydration can markedly weaken the equilibrium constraints imposed by the reverse reaction, thereby driving LA polycondensation toward higher conversion [52]. However, this tendency does not directly translate into lactide yield, since the subsequent depolymerization and ring-closure process remains kinetically controlled by intramolecular back-biting, product evaporation, and side reactions.

Mechanistically, the two-step process physically and operationally separates the molecular weight-building stage dominated by polycondensation from the molecular rearrangement stage governed by depolymerization and cyclization. This separation markedly reduces the risk of strong coupling among multiple reaction pathways, such as polycondensation, transesterification, racemization, and deep cracking, within a single reaction environment. This staged control strategy constitutes the core process principle by which the two-step route achieves high selectivity and high optical purity [46,53].

### 3.2. Process Characteristics

Based on the process principles outlined above, the two-step route exhibits a series of pronounced process characteristics under practical industrial operation, which is why it continues to be widely regarded as the most robust and mature industrial pathway for lactide production at present.

Its core advantage lies in the independent tunability of reaction conditions. The prepolymerization and depolymerization stages can be optimized separately with respect to temperature range, vacuum level, residence time, and catalyst selection, allowing chain growth, dehydration efficiency, and cyclization reactions to proceed within their respective optimal operating windows. This staged regulation markedly enhances reaction selectivity and product quality stability, and is particularly advantageous for the industrial synthesis of high optical-purity L-lactide [15,32].

Building on this basis, precise regulation of the oligomeric PLA structure enables the two-step process to effectively reduce the risk of cumulative amplification of side reactions. Studies have shown that the molecular weight, degree of polymerization, and composition of the prepolymer significantly affect the subsequent depolymerization behavior and the quality of crude lactide [54,55]. Shorter prepolymers generally have lower viscosity, which is favorable for heat transfer during the depolymerization stage and for the timely removal of lactide from the prepolymer phase. However, when the molecular weight of the prepolymer is excessively high, the increased system viscosity can limit mass transfer and make lactide evaporation more difficult [15,55]. Conversely, if the molecular weight of the prepolymer is too low or LA conversion is insufficient, residual LA and other chemical impurities may increase in the crude lactide product [55]. Therefore, in the two-step process, the oligomer structure is usually regulated by adjusting conditions such as polycondensation temperature, pressure, dehydration degree, and residence time, so as to achieve a balance among fluidity, thermal stability, and cyclization activity, thereby improving the selectivity of lactide formation via intramolecular back-biting. Meanwhile, strict dehydration can effectively suppress racemization during the depolymerization stage and prevent the configurational transformation of L-lactide into meso-lactide [32,56]. Further studies have shown that, under high-temperature conditions, the cyclic lactide structure itself may also undergo direct racemization, rather than relying solely on back-biting or transesterification processes occurring on oligomeric chain segments; this phenomenon is particularly pronounced during high-temperature depolymerization and distillation stages [57].

For catalytic systems, a key feature of the two-step process is that catalysts can be selected according to the distinct requirements of the prepolymerization and depolymerization stages. The prepolymerization stage places greater emphasis on the smooth progression of dehydration polycondensation and the control of prepolymer structure, whereas the depolymerization stage requires catalysts capable of efficiently promoting intramolecular back-biting to form lactide while minimizing racemization and side reactions under high-temperature conditions. Previous reviews have shown that tin-based compounds, such as SnO and Sn(Oct)_2_, have long been used as typical back-biting catalysts. Tin phosphite and rare-earth metal catalysts have also been employed to improve lactide formation kinetics, enhance catalytic stability, or regulate prepolymer molecular weight. However, different catalytic systems differ considerably in activity, stability, toxicity, and their tendency to induce racemization [15,32,55]. In addition, studies on the chemical recycling of PLLA have shown that Sn(Oct)_2_-catalyzed chain-end back-biting can selectively generate L-lactide at relatively low temperatures, providing useful insights into low-temperature catalytic depolymerization and lactide recovery [58].

From an engineering implementation standpoint, the two-step process is also more readily integrated with high-efficiency evaporation and reaction separation coupling units. The introduction of thin-film evaporators, wiped-film evaporators, and integrated distillation devices enables lactide to be rapidly removed from the high-temperature zone immediately after formation, thereby shifting the reaction equilibrium forward and suppressing stereochemical rearrangement and deep cracking [15,32,59]. This characteristic endows the two-step route with greater operability and long-term stability during continuous operation and scale-up.

Overall, the principal challenge of the two-step process does not lie in any individual reaction step, but rather in achieving precise coordination among thermal fields, mass-transfer pathways, and reaction kinetics at the industrial scale. Precisely because of its strong reliance on engineering design and process control, the two-step route remains irreplaceable in attaining high optical purity and high product consistency. This understanding has been comprehensively summarized and validated in recent systematic reviews and engineering analyses of industrial L-lactide production technologies [50,51,55].

### 3.3. Typical Catalyst Systems in Two-Step Lactide Synthesis

Catalysts determine the cyclization rate of oligomeric PLA, the temperature window for depolymerization, reaction selectivity, and the preservation of optical integrity, and thus constitute a key factor governing the overall performance of the two-step process.

#### 3.3.1. Tin-Based Catalysts

Traditionally, tin-based catalysts represented by stannous octoate (Sn(Oct)_2_) have been regarded as benchmark systems owing to their strong Lewis acidity and fast cyclization kinetics [15]. Full-process studies by Aliev et al. further confirmed their high activity in both oligomerization and depolymerization stages, while also indicating that, under high-temperature conditions, these catalysts readily induce charring and thermally driven racemization, leading to increased meso-lactide content [60].

These limitations have motivated a shift toward metal systems with milder Lewis acidity and lower toxicity, such as Zn- and Zr-based catalysts, in order to balance catalytic activity with optical retention.

#### 3.3.2. Mild Metal-Based Systems

In recent years, Zn-based catalysts have attracted particular attention. The ZnO creatinine composite system proposed by Tian et al. combines Lewis acid activation by ZnO with hydrogen-bond regulation by creatinine, enabling efficient cyclization while markedly suppressing racemization; the L-lactide produced in this system exhibits substantially higher optical purity than that obtained with conventional metal catalysts [61]. Similar behavior has also been observed in ZnO nanoparticle dispersion systems, where the relatively mild Lewis acidity of Zn species improves depolymerization efficiency and lactide formation under optimized reaction conditions [62]. Comparative studies further indicate that Zn-based catalysts exhibit favorable activity and selectivity for intramolecular transesterification of PLA oligomers, affording lactide in relatively high yield with low meso- and D,D-lactide formation, whereas Zr-based catalysts show moderate activity and somewhat higher stereoisomer formation [63]. By contrast, Al(III)-based catalysts display relatively weak activity and require higher temperatures and longer reaction times, while Ti(IV)-based catalysts may also promote further oligomer polymerization, thereby reducing lactide yield [63].

#### 3.3.3. Green Solid Acid and Metal Oxide Systems

Solid acids and metal oxide catalysts offer green and recyclable alternatives for two-step processes. Previous reviews have shown that solid or metal oxide-based systems, such as SnO, ZnO, tin phosphate, and rare-earth metal catalysts, can be applied to the depolymerization–cyclization of oligomeric PLA, showing certain potential in catalytic stability, molecular-weight regulation, and racemization control [15]. Among them, ZnO nanoparticle dispersion has been employed for the thermocatalytic depolymerization of oligomeric PLA to produce lactide. Tefara and Jiru systematically investigated the effects of reaction temperature, reaction time, and catalyst concentration on lactide yield, indicating that metal oxide nanocatalysts may serve as low-toxicity alternatives to conventional tin-based catalysts [62]. Aliev’s full-process study also suggested that catalyst selection should be considered in coordination with depolymerization temperature, product evaporation, and product purification, in order to avoid the accumulation of charring, racemization, and side reactions under high-temperature conditions [60].

#### 3.3.4. Composite or Bifunctional Catalyst Systems

Composite catalyst systems further improve the precision of reaction control. The Sn(II)/alcohol cocatalytic system reported by McGuire et al. stabilizes transition states via Sn-alkoxide intermediates, significantly enhancing cyclization selectivity while suppressing racemization [58]. The ZnO creatinine system exhibits a similar synergistic effect: ZnO activates ester bonds, while creatinine modulates the local polarity of the microenvironment, jointly lowering the energy barrier and enhancing enantioselectivity [61]. Current trends in catalyst design are therefore shifting from single Lewis acid centers toward cooperative systems based on “Lewis acid + hydrogen bonding” or “weakly acidic surfaces + confinement effects,” with the aim of achieving higher selectivity and improved optical stability.

Overall, although tin-based catalysts remain the benchmark in terms of catalytic activity for lactide synthesis, current research on two-step processes is increasingly moving toward alternative catalytic systems with improved selectivity and sustainability. In particular, three main development directions can be identified: mild metal-based systems, green solid acid and metal oxide catalysts, and composite or bifunctional catalytic systems. Compared with conventional tin catalysts, milder metal systems based on Zn and Zr exhibit clear advantages in preserving optical integrity and reducing racemization. Solid acid catalysts, on the other hand, offer promising prospects in terms of environmental friendliness, catalyst recyclability, and compatibility with continuous operation. Meanwhile, composite catalytic systems further enhance reaction control through cooperative effects and microenvironmental regulation, enabling improved cyclization selectivity and stereochemical retention. Together, these developments are promoting the evolution of the two-step route toward higher efficiency, lower racemization, and improved sustainability.

## 4. Comparative Analysis of One-Step and Two-Step Processes

The industrial routes for lactide production mainly comprise one-step and two-step processes, which exhibit pronounced differences in reaction pathways, catalyst requirements, energy consumption profiles, and their ability to ensure optical purity. A systematic comparison of these two approaches not only helps to clarify the key technical bottlenecks at the current stage but also provides a logical framework for subsequent process optimization and catalyst design.

### 4.1. Differences in Reaction Pathways and Mechanisms

The fundamental difference between the one-step and two-step processes is first reflected in the way the conversion pathway from LA to lactide is organized. As discussed above, the one-step process is represented by the LA → L_2_A → lactide pathway, emphasizing the direct dehydration–cyclization of LA into lactide via a dimeric intermediate under catalytic conditions, without involving an independent prepolymer preparation stage [37,38,39]. Therefore, the mechanistic key of this route does not lie merely in the formation of L_2_A, but rather in controlling the subsequent reaction direction of L_2_A. Specifically, L_2_A may either further cyclize to generate lactide or continue to condense with LA to form higher oligomers; meanwhile, the generated lactide may also undergo hydrolysis, thermally induced isomerization, or further side reactions. Accordingly, the selectivity of the one-step process is mainly determined by the rate balance between L_2_A cyclization and competing pathways such as oligomerization, hydrolysis, and isomerization [37].

By contrast, the two-step process follows a staged pathway of LA → oligomer/prepolymer → lactide, in which LA polycondensation and oligomeric PLA depolymerization–cyclization are relatively separated, allowing intermediates to be processed under different reaction conditions [15,32]. The first stage focuses on controlling the molecular weight and structure of the prepolymer, whereas the second stage promotes lactide formation through intramolecular back-biting, vacuum evaporation, and product removal. Since the prepolymerization and depolymerization stages can be independently optimized in terms of temperature, pressure, residence time, and catalyst selection, the two-step process can, to some extent, reduce mutual interference among polycondensation, depolymerization, racemization, and side reactions. Consequently, it exhibits higher process stability in the preparation of lactide with high optical purity [15,56,57].

Therefore, the difference between the two routes does not simply lie in the number of reaction steps, but rather in the different ways in which the fate of intermediates is controlled. In the one-step process, the formation, cyclization, and competing side reactions of L_2_A occur within the same reaction environment, making the process more sensitive to catalyst structure, water content, residence time, and product desorption behavior. By contrast, the two-step process separates intermediate structural regulation from the subsequent ring-closure reaction through the staged formation of prepolymers and their subsequent depolymerization, thereby providing a broader operational window for suppressing side reactions and controlling optical purity.

**Figure 6:**
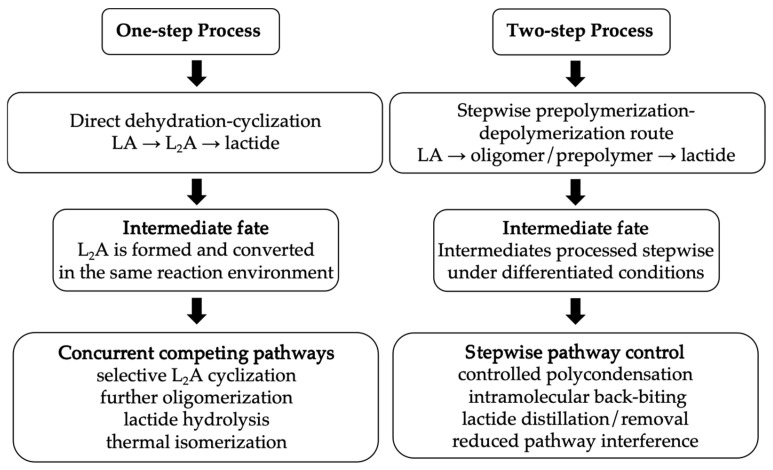
Structural comparison of reaction pathways in one-step and two-step lactide synthesis processes.

To provide an intuitive comparison of the two processes in terms of reaction pathways, intermediate fate, and competing reaction modes, Figure 6 structurally summarizes the organization of reaction pathways in the one-step and two-step routes.

### 4.2. Differences in Catalysts and Reaction Environments

The differences in catalyst requirements between the one-step and two-step processes mainly arise from the different ways in which the reaction processes are organized. In the one-step process, LA adsorption and activation, L_2_A formation and cyclization, suppression of further oligomerization, and lactide desorption all occur within the same reaction environment. Therefore, the catalyst is required not only to provide appropriate acidic sites, but also to regulate pore structure, diffusion pathways, and water adsorption behavior, so that the lactide-forming pathway can prevail over competing side-reaction pathways. By contrast, the two-step process separates prepolymerization from depolymerization–cyclization into two stages. The prepolymerization stage mainly controls LA polycondensation and prepolymer structure, whereas the depolymerization stage primarily promotes intramolecular back-biting and lactide evaporation. As a result, catalysts and reaction conditions can be optimized separately according to the requirements of different stages.

In one-step systems, catalyst design focuses on simultaneously promoting substrate activation, intermediate conversion, and product desorption. For Lewis acid catalysts, metal Lewis acid centers can interact with the carbonyl oxygen in LA or dimeric intermediates, thereby facilitating subsequent dehydration–cyclization. For example, open Sn sites in Sn-Beta zeolites are considered critical for enhancing catalytic activity, while adjacent Si-OH structures also influence substrate adsorption and the reaction energy barrier [43]. The SnO_2_-SiO_2_ gas-phase catalytic system further shows that an appropriate difference in the adsorption strength toward LA and lactide should be maintained: stronger adsorption of LA favors substrate activation, whereas weaker adsorption of lactide facilitates timely product desorption, thereby reducing further oligomerization or degradation [37].

For zeolitic solid acid systems, the accessibility of acidic sites, pore architecture, and diffusion behavior likewise influence the selectivity of the one-step process. Studies on ZSM-5 nanosheets have shown that reducing the b-axis thickness can enhance the accessibility of Brønsted acid sites and improve the diffusion of reactants and products within the pores, thereby favoring the conversion of L_2_A to lactide [38]. Further studies on hierarchical Beta zeolites indicate that crystal size, intercrystalline mesopores, and acid-site distribution jointly affect reactant diffusion, intermediate conversion, and product removal behavior in the one-step conversion of LA [64]. In addition, organic framework catalysts such as COFs also demonstrate that regular pore structures and functional groups can cooperatively influence the dimerization and cyclization of LA molecules. For example, functional sites such as carboxyl and ketone groups in OH-COOH-COF can promote the conversion of L-LA to L-lactide through hydrogen-bonding interactions and pore confinement [45]. Therefore, the key to catalyst design in the one-step process is not simply to increase acid strength, but rather to achieve an appropriate balance among acidic sites, pore structure, diffusion behavior, water adsorption, and product desorption.

By contrast, the catalytic functions in the two-step process exhibit more distinct stage-specific characteristics. The prepolymerization stage mainly focuses on LA dehydration polycondensation and prepolymer structure control, whereas the depolymerization stage requires catalysts to promote intramolecular back-biting of oligomeric PLA to generate lactide while minimizing racemization, transesterification, and thermal degradation under high-temperature conditions. Previous studies have shown that tin-based compounds such as SnO and Sn(Oct)_2_ have long been used as typical back-biting catalysts, owing to their relatively high activity and their ability to participate effectively in the depolymerization process within polymer melts. However, under high-temperature depolymerization conditions, tin-based catalytic systems may also induce charring, racemization, and increased meso-lactide content. Therefore, different catalytic systems exhibit distinct differences in activity, stability, toxicity, and racemization tendency [15,32,60].

Further process optimization studies have shown that temperature, pressure, catalyst type, and catalyst loading jointly influence the lactide formation rate, crude product purity, and degree of racemization. Therefore, catalyst selection in the two-step process should be considered in coordination with depolymerization temperature, vacuum conditions, and product evaporation efficiency [55]. In recent years, the Zn(La)_2_/creatinine composite catalytic system has also reflected the strategy of catalyst composition optimization in the two-step process. Zn(La)_2_ can promote LA polycondensation and the depolymerization–cyclization of oligomers, while the introduction of creatinine helps suppress racemization and improve the optical purity of the product. These results indicate that although the two-step process can reduce mutual interference among reaction pathways through stage separation, the depolymerization stage still requires a balance among back-biting activity, optical stability, and product evaporation [65].

As shown in Table 2, tin-based catalysts generally exhibit high activity but may cause racemization, side reactions, and metal residue issues under high-temperature conditions. In contrast, zinc-based coordination systems are more favorable for optical purity control, although further optimization of catalyst composition and operating conditions is still required.

Overall, the differences between the one-step and two-step processes in terms of catalysts and reaction environments do not lie in whether one specific type of catalyst is absolutely superior, but rather in the different reaction tasks that the catalyst is required to perform. In the one-step process, the catalyst must simultaneously regulate LA activation, L_2_A cyclization, side-reaction suppression, and product desorption within the same reaction environment, making the process more sensitive to acidic sites, pore architecture, and diffusion behavior. By contrast, the two-step process separates the prepolymerization and depolymerization stages, allowing catalysts and reaction conditions to be adjusted according to the specific requirements of each stage. This provides a broader regulatory window for suppressing racemization, improving product purity, and enhancing process stability.

### 4.3. Engineering Trade-Offs Among Energy Efficiency, Selectivity, and Optical Purity

From the perspective of process control, the one-step and two-step processes exhibit different characteristics in terms of energy consumption, selectivity, and optical purity control. Since the one-step process does not involve independent prepolymer preparation and depolymerization stages, it has potential advantages in process simplification, reduced material transfer, and continuous operation. Particularly in gas-phase one-step systems, LA is preheated and vaporized before entering a fixed-bed reactor, where it is directly converted into lactide over a solid catalyst. In principle, this route can reduce the energy consumption associated with high-temperature, high-vacuum depolymerization and multistage separation in conventional two-step processes [37,44,66].

However, the advantages of the one-step process are not unconditional. Since the target pathway of LA → L_2_A → lactide and side reactions such as oligomerization, hydrolysis, and thermally induced isomerization occur within the same reaction environment, its selectivity and optical purity are highly sensitive to reaction temperature, water content, residence time, catalyst surface adsorption/desorption behavior, and crude lactide purity [37,44].

In gas-phase one-step processes, continuous operation and short residence time are important strategies for improving selectivity. The SnO_2_-SiO_2_ catalytic system demonstrates that, when the catalyst can effectively adsorb LA while promoting timely desorption of lactide, the continued oligomerization or degradation of the product on high-temperature catalytic surfaces can be reduced [37]. Continuous process design based on this type of system further indicates that proper matching among rapid reaction, product quenching, and subsequent crystallization/purification processes is essential for maintaining lactide yield and product quality [66]. Techno-economic and life-cycle analyses also show that the gas-phase one-step process offers certain advantages in terms of conversion cost and global warming potential; however, its overall performance is highly dependent on the operating conditions of the reaction section. In particular, reaction temperature and crude lactide purity can significantly affect the downstream purification burden and process economics [44]. Therefore, process intensification in the one-step route should focus on short residence time, rapid product removal, and effective reaction–separation integration, so as to mitigate the adverse effects of multipathway competition within the same reaction environment on selectivity and optical purity.

By contrast, the energy consumption of the two-step process mainly arises from multiple unit operations, including LA concentration, prepolymer formation, low-pressure depolymerization, and subsequent lactide purification. This route generally requires continuous dehydration during the prepolymerization stage to drive the polycondensation reaction, followed by high-temperature and reduced-pressure conditions during the depolymerization stage to promote intramolecular back-biting and lactide evaporation. Therefore, the overall process is relatively long, with higher requirements for equipment and energy input [15,32].

However, the advantage of the two-step process lies in the fact that the prepolymerization and depolymerization stages can be regulated separately. During the prepolymerization stage, the structure of oligomeric PLA can be controlled by adjusting LA concentration, water content, polycondensation temperature, and residence time. During the depolymerization stage, intramolecular back-biting, racemization, and thermal degradation can be regulated through temperature, vacuum level, catalyst type, and product evaporation efficiency [15,32,55,67]. For example, the vacuum level directly affects the formation and evaporation behavior of crude lactide, whereas LA feed concentration and water content influence the composition of oligomers and their subsequent depolymerization–cyclization process [67,68]. Therefore, although the two-step process involves a more complex flowsheet and higher energy consumption, its staged regulation strategy is favorable for achieving a more reliable balance among high yield, low racemization, and product stability.

Optical purity control is a key issue shared by both routes, although the sources of risk differ. In the one-step process, water, heat, and product residence time directly affect the risks of hydrolysis and isomerization of the generated lactide. Therefore, efficient water removal, short residence time, and rapid product desorption/condensation are required to minimize side reactions [37,44]. In the two-step process, racemization is mainly associated with high-temperature exposure during the prepolymerization and depolymerization stages, residual water, catalyst type, and product evaporation efficiency. Relevant studies have shown that water not only promotes hydrolysis, but may also participate in lactide racemization; meanwhile, L-lactide itself may undergo direct racemization or isomerization under high-temperature conditions. Therefore, in the two-step process, strict dehydration, reduced high-temperature residence time, timely evaporation of lactide, and the selection of low-racemization catalytic systems are important strategies for maintaining optical purity [55,56,57]. Studies on oligomer depolymerization and composite catalytic systems further indicate that optimization of catalyst composition and rapid product removal can, to some extent, improve lactide yield and reduce the risk of racemization [51,65,69].

Process intensification provides a common direction for optimizing both routes, although their intensification priorities are not the same. For the one-step process, process intensification mainly focuses on continuous gas-phase reactors, short residence-time control, rapid product condensation, and effective reaction–separation integration, with the aim of reducing selectivity losses caused by multipathway competition within the same reaction environment [37,44,66]. For the two-step process, process intensification is more centered on prepolymer structure control, low-pressure depolymerization, thin-film/wiped-film evaporation, reaction–separation coupling, and efficient distillation. The purpose is to improve heat and mass transfer efficiency, shorten the residence time of lactide in high-temperature zones, and reduce the risks of racemization, repolymerization, and deep degradation [15,32,59,68]. From the general principles of process intensification, reaction–separation coupling, continuous operation, and enhanced heat and mass transfer are all beneficial for reducing energy consumption, suppressing side reactions, and improving product quality. However, the specific technical route still needs to be matched with the reaction phase state, feedstock composition, catalyst stability, and separation load [70].

Overall, the one-step and two-step processes exhibit different priorities in terms of energy consumption, selectivity, and optical purity control. The one-step process is advantageous due to its shorter flowsheet and higher potential for continuous operation, and may outperform the conventional two-step process in terms of conversion cost and environmental impact. However, its selectivity and product purity are highly dependent on the catalyst surface environment, reaction residence time, water content, and crude lactide purity [37,44,66]. By contrast, although the two-step process involves a longer flowsheet and higher energy consumption, the separation of prepolymerization and depolymerization stages allows the oligomer structure, back-biting reaction, and lactide evaporation to be regulated independently. Therefore, it still exhibits strong advantages in achieving high optical purity and long-term stable operation [15,32,55]. Accordingly, the two routes should not be regarded as simply substitutive. The one-step process is more suitable for development toward low energy consumption, continuous operation, and process integration, whereas the two-step process remains a more mature and controllable technological route for the current industrial production of high-purity lactide.

### 4.4. Differences in Kinetic Behavior and Process Control

Lactide synthesis involves multiple processes, including LA dehydration, dimer or oligomer formation, cyclization, product evaporation, and suppression of side reactions. Therefore, its macroscopic rate behavior is often not determined by a single chemical reaction step alone, but is also influenced by water removal, mass transfer, system viscosity, catalyst activity, residence time, and product desorption efficiency. In this context, the significance of kinetic analysis is not limited to obtaining a specific reaction rate constant, but rather lies in helping to understand the competitive relationships among the target reaction, product selectivity, and side reactions under different operating conditions.

For the one-step process, the core of kinetic control is to promote the further dehydration–cyclization of LA through a dimeric intermediate to form lactide within a short residence time, while suppressing competing pathways such as oligomerization, hydrolysis, and thermally induced isomerization. Studies on the SnO_2_-SiO_2_ gas-phase one-step process have shown that stronger adsorption of LA on the catalyst is favorable for substrate activation, whereas weaker adsorption of lactide facilitates timely product desorption, thereby reducing further oligomerization or degradation. Therefore, the adsorption/desorption behavior on the catalyst surface represents an important kinetic factor affecting the selectivity of the one-step process [37]. In continuous gas-phase process design, the matching among reaction residence time, product quenching, and subsequent crystallization/purification processes also affects the final yield and product quality, indicating that kinetic analysis of the one-step process should be integrated with continuous reactor design and process simulation [66].

By contrast, the kinetic behavior of the two-step process is more stage-dependent. The prepolymerization stage mainly involves LA dehydration polycondensation and the formation of oligomeric PLA structures, and its rate is influenced by factors such as the initial LA concentration, water content, degree of dehydration, and reaction time. Harshe et al. established a dynamic mathematical model for LA polycondensation, using conversion, changes in water concentration, and molecular-weight distribution as the main outputs, while incorporating water volatilization and removal into the kinetic description. This indicates that water removal and mass-transfer behavior are important factors for understanding rate variations during the prepolymerization stage [52]. Related studies further show that oligomer compositions differ among LA systems with different concentrations, while water content affects the polycondensation equilibrium and the subsequent lactide formation process. Therefore, kinetic analysis of the prepolymerization stage should be considered together with feed composition and water removal behavior [67].

The depolymerization stage mainly involves competing processes such as intramolecular back-biting of oligomers, lactide evaporation, racemization, and thermal degradation. Among these factors, the vacuum degree directly affects the formation and evaporation behavior of crude lactide, while excessively low or otherwise inappropriate pressure conditions may also alter the balance between product evaporation and side reactions [68].

Further analysis shows that temperature, pressure, catalyst type, and catalyst loading jointly influence the lactide formation rate, crude product purity, and degree of racemization. Therefore, the kinetic behavior in the two-step process cannot be explained solely by a single reaction step, but should instead be comprehensively analyzed by considering prepolymer molecular weight, system viscosity, depolymerization temperature, vacuum degree, and product removal efficiency [55]. For example, in the Zn(La)_2_/creatinine composite catalytic system, the authors conducted kinetic analysis of LA polycondensation and subsequent lactide formation, and applied a pseudo-second-order kinetic model for apparent fitting of the related processes [65]. The significance of this model lies mainly in describing the overall rate variation in polycondensation or oligomer formation under specific reaction conditions, rather than directly proving a particular elementary reaction step. Therefore, the pseudo-second-order kinetic model is more appropriately understood as an apparent descriptive method for specific systems, with its parameters reflecting the combined effects of reaction conditions, catalyst function, and mass-transfer factors.

Overall, kinetic analysis has different interpretative meanings in the one-step and two-step processes. The one-step process places greater emphasis on the competition among LA activation, L_2_A cyclization, product desorption, and side reactions within a short residence time. By contrast, the two-step process focuses more on the stage-specific matching among prepolymer formation, intramolecular back-biting, and lactide evaporation. Therefore, kinetic models should be regarded as auxiliary tools for process control and scale-up, rather than as universal mechanistic criteria applicable to all reaction systems. Their rational use can help explain the differences between the two routes in terms of operational stability, selectivity, and optical purity control.

### 4.5. Industrial Compatibility and Route Positioning

From the perspective of industrial compatibility, the one-step and two-step processes correspond to different process objectives and application scenarios, and therefore should not be regarded as simply substitutive. The main appeal of the one-step process lies in its simplified flowsheet and potential for continuous operation. Particularly in gas-phase one-step systems, LA is preheated and vaporized before entering a fixed-bed reactor, where it is directly converted into lactide over a heterogeneous catalyst. This can reduce the process complexity associated with independent prepolymerization, depolymerization, and intermediate material transfer in the conventional two-step route. The techno-economic and life-cycle analyses conducted by Heo et al. based on a one-step gas-phase route showed that this process has certain advantages in terms of conversion cost and global warming potential. However, its overall performance is highly dependent on reaction temperature, crude lactide purity, and the downstream purification burden [44].

Regarding process scale-up, whether the one-step process can achieve stable industrial operation depends not only on the reduction in reaction steps, but also on the coordinated matching among continuous reactor design, catalyst lifetime, and product separation. Studies on continuous gas-phase process design have shown that short residence time, product quenching, and effective integration with subsequent crystallization/purification processes are important for maintaining high lactide yield and product quality [66]. Meanwhile, the SnO_2_-SiO_2_ catalytic system exhibits high selectivity and long-term stability under continuous atmospheric-pressure conditions, indicating that stable heterogeneous catalysts constitute an important foundation for advancing the one-step process toward continuous and large-scale production [37]. Nevertheless, overall, the one-step process is currently more suitable as a process direction with potential for low energy consumption and continuous operation, while its industrial application still requires further solutions to issues such as fluctuations in crude product purity, long-term catalyst stability, and separation burden.

By contrast, the two-step process remains the more mature and controllable technological route in current industrial lactide production. Its advantages arise from the separation of the prepolymerization and depolymerization stages. In the prepolymerization stage, the molecular weight and structure of oligomeric PLA can be regulated, whereas in the depolymerization stage, high-purity lactide can be obtained through catalytic intramolecular back-biting, reduced-pressure evaporation, and subsequent refining [15]. Although this route involves a longer flowsheet and is accompanied by energy burdens associated with LA concentration, prepolymer formation, high-temperature low-pressure depolymerization, and rectification purification, its process window is relatively clear and its product quality stability is high. Therefore, it remains suitable for the large-scale preparation of high-optical-purity L-lactide or D-lactide [32].

The two-step process also exhibits strong compatibility with the downstream PLA industrial chain. The preparation of high-molecular-weight PLA generally relies on ROP of high-purity lactide; therefore, the chemical purity and optical purity of lactide directly affect the molecular weight, stereoregularity, thermal properties, and application scope of the final PLA products [71,72]. The full-cycle laboratory-scale technology reported by Aliev et al. further demonstrates an integrated process from LA concentration, oligomerization, lactide synthesis and purification to subsequent ROP and PLA pellet preparation, indicating that lactide purification and quality control play a critical role in the process chain from LA to PLA [60]. This also explains why the two-step process, despite its greater process complexity, still shows strong practical compatibility in the production of high-quality PLA.

From the perspective of specific process optimization, future improvements in the two-step process do not necessarily require changes to its basic route. Instead, continuous optimization should focus on the issues of energy consumption, racemization, and product stability within the existing flowsheet. Temperature, pressure, catalyst type, and catalyst loading can influence the lactide formation rate, crude product purity, and degree of racemization; therefore, depolymerization conditions and product evaporation efficiency remain key factors affecting the industrial stability of the two-step process [55]. Meanwhile, vacuum degree and LA feed concentration also affect crude lactide formation, oligomer composition, and the subsequent depolymerization–cyclization behavior, indicating that optimization of the two-step process should still begin with the coordinated matching among feedstock pretreatment, prepolymer structure control, and the depolymerization-evaporation process [67,68].

Overall, the industrial positioning of the one-step and two-step processes differs significantly, but this comparison should be understood more appropriately as a trend-level judgment based on representative systems, rather than as a fully universal conclusion. The two-step process remains the more mature and controllable route for large-scale production at present. Although the one-step process has potential advantages in flowsheet simplification, continuous operation, and reduced energy consumption, its practical performance is highly dependent on catalyst structure, reactor design, water removal efficiency, product desorption, and control of crude lactide purity. Therefore, the applicability of the two routes should be evaluated in relation to specific catalytic systems and process boundaries, rather than through a simple ranking of superiority.

Based on the above discussion, Table 3 summarizes the differences between the one-step and two-step processes across key technological dimensions.

## 5. Future Perspectives

With the continued expansion of the PLA industry and the deepening implementation of sustainable chemistry principles, research on lactide preparation is gradually shifting from a sole pursuit of high conversion and high yield toward the synergistic optimization of green catalysis, low-energy processes, high optical-purity control, and integration with downstream polymerization and recycling systems. As the key monomer linking LA to high-molecular-weight PLA, the chemical purity, optical purity, water content, acidic impurities, and metal residues of lactide can all affect the subsequent ROP process, as well as the molecular weight, crystallization behavior, thermal properties, and application scope of the final PLA products. Therefore, future lactide preparation processes need to be systematically optimized at multiple levels, including catalysts, feedstocks, process engineering, monomer quality, and recycling [73,74].

First, green, low-toxicity, and recyclable catalytic systems remain an important direction for future development. Although traditional metal catalytic systems generally exhibit high activity, issues such as metal residues, difficulties in catalyst recovery, and compatibility with subsequent polymerization still need to be addressed. For the one-step process, heterogeneous solid acids and confinement catalytic materials show considerable potential. For example, a Si-doped γ-Al_2_O_3_ confinement catalytic system can promote the direct conversion of L-LA to L-lactide by regulating pore structure and the acidic environment, demonstrating the application prospects of low-cost solid oxide catalysts in one-step synthesis [75]. Brønsted acid-catalyzed one-step processes also show the possibility of reducing metal residues and achieving relatively mild reaction conditions [76]. In addition, studies on Al-IPC zeolites indicate that the matching among pore size, acid type, and acid-site density affects the selectivity toward L_2_A and L-lactide. This suggests that future catalyst design should place greater emphasis on the relationship among pore structure, acidic sites, and reaction pathways, rather than simply increasing catalytic activity [77].

Secondly, the greening of feedstock sources and life-cycle optimization will become important components of the sustainable development of lactide preparation. The use of agricultural by-products, non-food biomass, or other low-value carbon sources to produce LA, followed by its further conversion into lactide, can help reduce both the feedstock cost and environmental burden of the PLA value chain [78]. Studies on lactide preparation from fermentation-derived LA obtained from agricultural sources have shown that low-cost biomass-derived LA can serve as a potential feedstock. However, the effects of impurities, water, salts, and organic acids on subsequent lactide synthesis and purification still require further evaluation [79]. Life-cycle assessment studies have also indicated that the environmental performance of PLA does not depend solely on the polymer itself; instead, feedstock production, fermentation, monomer preparation, and post-treatment processes all contribute to its overall environmental burden. Therefore, future lactide processes should be developed in coordination with low-environmental-impact feedstocks and low-energy process technologies [80].

In addition to conventional routes using LA as the direct feedstock, lactate esters also provide a new feedstock pathway for future lactide preparation. Lactate esters can generally serve as intermediates in LA purification processes and may also be obtained from renewable sugars or other feedstocks through chemocatalytic routes. Therefore, the direct preparation of lactide from lactate esters is expected to reduce steps such as LA hydrolysis and redistillation. De Clercq et al. proposed a continuous gas-phase transesterification route using alkyl lactates as feedstocks, achieving the selective conversion of methyl lactate to lactide over a TiO_2_/SiO_2_ catalyst, and further pointed out the recycling potential of unreacted lactate esters and linear dimer esters [28]. Subsequent studies on Ti-MWW zeolites further demonstrated that the dispersion of Ti sites, open pore structure, and external surface accessibility significantly influence lactide selectivity and productivity during methyl lactate conversion [81]. In addition, Ai et al. developed a separation-promoted liquid-phase self-transesterification strategy, in which the timely removal of alcohol by-products weakens thermodynamic limitations, providing another approach for process intensification of lactate ester-based routes [82]. However, since this type of route uses lactate esters rather than LA as the feedstock, its reaction mechanism, equilibrium constraints, and separation requirements differ from those of the LA-based one-step process discussed in this review. Therefore, it is more appropriately considered as a future direction for alternative feedstocks and process integration.

Beyond the strictly defined one-step and two-step processes, hybrid processes may also be considered as a future direction for process intensification. Such processes do not simply combine the two routes, but rather attempt to achieve moderate staged regulation and continuous coupling of key steps, including dehydration, cyclization, product removal, and purification, while avoiding excessive process complexity associated with conventional two-step routes. In principle, this design may establish a new balance among process compactness, reaction selectivity, and process controllability. However, its practical effectiveness still depends on the catalytic system, reactor design, water removal efficiency, crude lactide purity, and downstream separation burden. Further studies combining reaction kinetics, separation load, and techno-economic evaluation are still required to clarify its applicable boundaries.

At the process-engineering level, future lactide preparation should not be optimized solely around a single reaction step, but should place greater emphasis on the overall integration of reaction, separation, purification, and waste-stream treatment. For monomers such as lactide, which are highly sensitive to water, acidic impurities, and high-temperature residence time, the key to process scale-up lies not only in improving reaction conversion, but also in stabilizing crude product composition, reducing the downstream purification burden, and minimizing by-product accumulation. Concepts from sustainable polymer research and process intensification also suggest that future monomer preparation processes should incorporate energy consumption, water consumption, solvent use, waste-stream treatment, and life-cycle performance into comprehensive evaluation, rather than relying primarily on laboratory-scale yield as the main criterion [73,83].

Meanwhile, future lactide preparation must be more closely aligned with the monomer-quality requirements of downstream ROP. Industrial lactide is subject to strict specifications regarding free acidity, water content, metal ions, and stereochemical purity. Among these factors, water and acidic impurities can limit the attainable molecular weight of PLA, metal ions may induce hydrolysis, oxidation, or racemization, and stereochemical purity directly affects the melting point, crystallinity, and mechanical properties of PLA [74]. Therefore, future lactide processes should not focus solely on achieving high crude yields, but should also place greater emphasis on controlling impurities such as residual LA, oligomers, water, meso-lactide, and catalyst residues. ROP-related studies have shown that catalyst structure, metal centers, ligand environment, and monomer purity can influence polymerization rate, molecular weight, dispersity, and stereochemical control. Studies on functionalized lactide copolymerization systems further indicate that lactide monomer quality can also affect the structure and properties of functionalized PLA materials [34,84,85].

Finally, lactide preparation should also be integrated with PLA chemical recycling and closed-loop utilization. A sustainable PLA value chain in the future requires not only the efficient production of high-purity lactide from LA, but also the development of recycling pathways that convert waste PLA back into lactide or LA. Reviews on chemically recyclable polymers have pointed out that reversible polymer–monomer conversion is an important direction for achieving closed-loop plastic utilization, with the chemical recycling of polyester materials receiving particular attention [86]. Meanwhile, studies on the synthesis and chemical transformation of lactide and glycolide have shown that lactide is not only the monomer for PLA polymerization, but can also participate in hydrolysis, alcoholysis, epimerization, and pyrolysis reactions. This provides broader possibilities for by-product treatment, reutilization of low-purity lactide, and closed-loop PLA recycling [87].

Overall, future advances in lactide preparation should proceed through the coordinated development of green catalysis, low-carbon feedstocks, continuous processes, monomer quality control, and closed-loop recycling. Only by integrating catalyst design, process engineering, downstream polymerization requirements, and life-cycle assessment can the economic viability, sustainability, and industrial competitiveness of lactide production technologies be further improved.

## 6. Conclusions

In summary, the one-step and two-step processes for lactide preparation each exhibit distinct characteristics and have continued to evolve in recent years through advances in mechanistic understanding, catalyst design, and process intensification. The one-step process is centered on the direct dehydration–cyclization of LA to lactide via a dimeric intermediate. It offers potential advantages in flowsheet simplification and continuous operation, but its selectivity is highly dependent on catalyst structure, water content, residence time, and product desorption behavior. By contrast, the two-step process relies on staged control of prepolymerization and depolymerization–cyclization, providing higher process stability in prepolymer structure regulation, intramolecular back-biting selectivity, product evaporation, and optical purity retention. Therefore, it remains the more mature technological route for the current industrial preparation of high-purity lactide.

Nevertheless, existing processes still face challenges in side-reaction control, energy-consumption reduction, and stable industrial scale-up. These challenges may also lead to the presence of residual water, unreacted LA, meso-lactide, and low-molecular-weight oligomeric species in the final lactide product, thereby affecting its chemical purity, optical purity, and subsequent ROP performance. For the one-step process, further efforts are needed to improve selectivity control between the direct cyclization pathway and competing reactions such as oligomerization, hydrolysis, and isomerization. For the two-step process, it remains necessary to reduce the energy consumption and separation burden associated with concentration, prepolymerization, depolymerization, and purification while maintaining high optical purity. Future research should focus on green and recyclable catalysts, regulation of pore structures and acidic sites, continuous reactors, reaction–separation coupling, and ROP-oriented monomer quality control. By integrating catalyst design, process engineering, and the performance requirements of downstream PLA, lactide preparation technologies are expected to further advance toward greener, more efficient, and scalable production.

## Figures and Tables

**Table 1:** Standardized comparison of representative catalyst systems for one-step lactide synthesis.

Catalyst	Yield	Selectivity	Optical Purity	Reaction Condition	Reference
H-Beta	84.7% with CaCl_2_; 72.3% without salt	Not reported.	Not reported.	Liquid phase, toluene, 140 °C, 3–9 h	[40]
F-modified H-Beta	84 mol%; H-beta: 70 mol%	Not reported.	Not reported.	Liquid phase, toluene, 140 °C, 3 h	[42]
ZSM-5 nanosheets	60.5%	Not reported.	Not reported.	Liquid phase, toluene, oil bath 140 °C, 110 °C actual reaction temperature, 5 h	[38]
Sn-Beta	33.95%	Product distribution: LA 11.09%, L_2_A 18.47%, L_m_A 36.49%	L-LT 99.70%; meso-LT 0.30%	Mesitylene, oil bath 180 °C, 20 min	[43]
SnO_2_-SiO_2_	93–94%	High LT selectivity; low oligomer formation	Almost 100% enantioselectivity	Gas-phase fixed bed, atmospheric pressure, 240 °C, WHSV 1.0 h^−1^	[37]
COFs	80–81%	L-lactide/LA-oligomers ratio = 5:1	meso-lactide ≈ 1%; little or no isomerisation	Liquid phase, toluene, 140 °C, 3.5 h	[45]

**Table 2:** Comparison of representative catalytic systems for lactide synthesis.

Catalytic System	Main Advantages	Effect on Purity/Stereoselectivity	Operating Conditions and Limitations	References
Sn(Oct)_2_, SnCl_2_, and other tin-based catalysts	High activity; promote oligomer depolymerization and intramolecular back-biting	Increase lactide formation rate, but may cause racemization and side reactions at high temperatures	Commonly used under high-temperature and reduced-pressure conditions; possible metal residue and toxicity issues	[15,32,60]
Zn(La)_2_/creatinine zinc-based coordination system	Mild Lewis acidity; hydrogen-bonding/coordination-assisted cyclization	Reduces racemization and improves L-lactide optical purity	Catalyst composition, temperature, vacuum degree, and product evaporation still require optimization	[65]

**Table 3:** Comparison of key characteristics between one-step and two-step lactide synthesis routes.

Comparison Aspect	One-Step Route	Two-Step Route
Basic pathway	LA → L_2_A → lactide	LA → oligomer/prepolymer → lactide
Intermediate control	L_2_A forms and converts within the same reaction environment	Prepolymers can be regulated before depolymerization
Main side reactions	Oligomerization, hydrolysis, thermally induced isomerization	Racemization, transesterification, thermal degradation, repolymerization
Catalyst requirements	Must simultaneously promote LA/L_2_A activation, cyclization, and product desorption	Catalysts can be selected separately for the prepolymerization and depolymerization stages
Sensitivity to reaction environment	Sensitive to water content, residence time, and desorption behavior	Sensitive to prepolymer structure, depolymerization temperature, and vacuum degree
Energy consumption and separation	Shorter flowsheet with potential for lower energy consumption, but dependent on crude product purity	Longer flowsheet with more separation/purification units, but more mature process control
Optical purity control	Requires water-content control and shortened high-temperature residence time of the product	Requires control of residual water, high-temperature depolymerization time, and product evaporation
Compatibility with continuous operation	More readily designed as a continuous gas-phase process	Can also be operated continuously, but involves more process units
Industrial positioning	Has potential for flowsheet simplification and greener production	Currently more mature and suitable for large-scale production of high-purity lactide

## Data Availability

No new data were created or analyzed in this study. Data sharing is not applicable.
